# The Role of Early Rehabilitation in Better Outcomes in a Rare Presentation of Tuberculous Meningitis With Broca’s Aphasia

**DOI:** 10.7759/cureus.53793

**Published:** 2024-02-07

**Authors:** Ghanishtha C Burile, Pallavi Harjpal, Neha P Arya, Nikita H Seth

**Affiliations:** 1 Neurophysiotherapy, Ravi Nair Physiotherapy College, Datta Meghe Institute of Higher Education and Research, Wardha, IND

**Keywords:** speech-language therapy, tubercular meningitis, recurrent low-frequency transcranial magnetic stimulation, transcranial direct current stimulation, medical therapy, broca's aphasia

## Abstract

There is a complex link between tuberculous meningitis (TBM) and aphasia, in which a language impairment is caused by an injury to the cortical language centre. The parts of the brain that function for speech and language production are the Wernicke's, Broca's, and arcuate fasciculus regions. This case report mainly highlights the neurological consequences of TBM, and how it affects language and speech functioning. It outlines a comprehensive physiotherapy rehabilitation program that targets a range of issues for the patient, such as verbal output, weakness, motor deficits, articulation issues in speech, and coordination issues. Various treatment modalities can help correct weakness, improve balance and coordination, increase flexibility and range of motion (ROM), and make speech more fluent. The case report emphasizes the necessity of using an integrated approach that combines speech-language therapy (SLT), melodic intonation therapy (MIT), constraint-induced aphasia therapy (CIAT), medication treatments, and physical therapy to address the multifaceted impacts of TBM-induced aphasia on a patient's quality of life (QOL).

## Introduction

Schutz recorded the early documentation of an instance of aphasia caused by tuberculous meningitis (TBM) in 1881. Mycobacterium tuberculosis (MTB) is still a common central nervous system infection source, causing a broad spectrum of clinical symptoms [1]. Aphasia occurs when an injury to a cortical language centre induces a loss of language understanding or formulation. Typically, language centres are situated in the dominant hemisphere of the brain. These structures illustrate the arcuate fasciculus, Broca's area, and Wernicke's area. It serves as a centre for word comprehension and planning by fusing audio and visual data. The Broca's area is situated in the inferior frontal brain and is responsible for muscular speech execution and sentence construction. The arcuate fasciculus is the neural pathway that connects the Wernicke's and Broca's areas [2]. Strokes frequently induce aphasias, but brain traumas, brain tumours, and other pathological conditions such as neurodegenerative diseases and dementia can also cause them.

Broca's, Wernicke's, global, and transcortical aphasias are the most prevalent forms of aphasia; conduction aphasia has been observed in cortical injury instances without subcortical extension. Also, Wernicke's and motor/premotor frontal areas are connected by the arcuate fasciculus rather than Wernicke's and Broca's areas. Patients with conduction aphasia may take weeks to months to recover. Conduction aphasia differs from Wernicke's aphasia and Broca's aphasia in that there is an isolated inability to repeat. Therefore, it is necessary to plan rehabilitation protocols to get better outcomes [3]. Aphasia is an impairment that affects 21-38% of stroke survivors. The annual community incidence is 43/100,000, and the prevalence is 3000 per million [4]. The right-sided lateralization of the Broca's area is caused by a nidus near the Broca's region in patients with cerebral arteriovenous malformations (AVMs). Conversely, a nidus near the Wernicke's area is responsible for the right-sided lateralization of the Wernicke's region [5]. Benson and Geshwind categorize aphasic patients into two groups, which are aphasia without repetitive disorders and aphasia with repetitive disorders [6]. The most common aetiology for non-fluent aphasia is a lesion impacting Broca's area in the left posterior inferior frontal region of the left frontal lobe [7].

In recent years, language and speech processing have also been linked to several additional brain regions, including the subcortical structures known as the basal ganglia and the right and left hemispheres of the cerebral cortex [8]. A neuroscience-inspired approach to aphasia therapy allows the physician to start thinking about intervention at the neuronal assembly level and viewing therapy insights into other methods that can be used to improve learning. One area for improvement is the errorless learning process. In an errorless learning activity, the learner's chances of making mistakes are lowered or eliminated [9]. Since intensive, comprehensive aphasia programs (ICAPs) address many areas through distinct treatment methodologies and formats, they are different from programs that offer a single treatment very intensely, like constraint-induced language therapy [10]. The benefits of transcranial magnetic stimulation alone are insufficient. Although speech and language therapy alone is effective in naming ability, transcranial magnetic stimulation in addition to speech and language therapy significantly increases the gain obtained with therapies [11]. In our case, there was an acute infarct in the left caudate. The relationship between TBM and Broca's aphasia is a typical complication in these patients with TBM. Therefore, an integrated physiotherapy treatment plan, including speech therapy, should be planned to improve the patient's Quality of Life (QoL) [12].

## Case presentation

**Figure 1:**
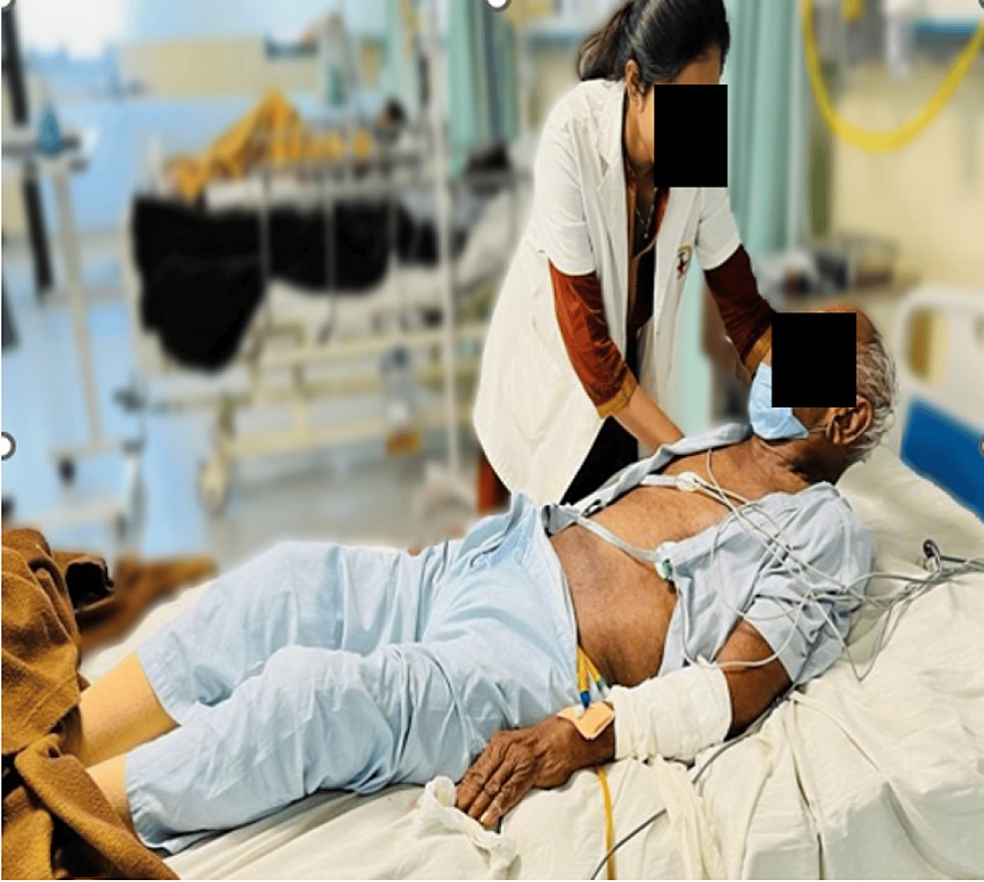
Brudzinski's sign was positive indicating neck rigidity; passive flexion of the neck caused slight flexion of both legs and thigh

A 73-year-old male patient visited a tertiary care hospital in the emergency department at Sawangi, Wardha, India, with the chief complaints of headache, fever, and blurred vision, leading to a fall in the bathroom. He was taken to a local practitioner, where medications were given, and he got relief. After four days, he had slurred speech, generalized weakness, multiple episodes of diarrhoea, reduced food intake, headache, generalized seizures, visual disturbances, sensory deficits, and behavioural changes. For these complaints, he was again brought to the hospital, where a neurological examination, which included normal reflexes and tone assessment, indicated hypotonia. Signs of TBM (Brudzinski's sign) were positive, indicating neck rigidity; passive neck flexion caused a slight flexion of both legs and thigh, as depicted in Figure 1. The diagnostic investigation included an MRI of the brain. The assessment of reflexes has been mentioned in Table 1.

**Table 1:** Reflexes assessment +: Diminished, ++: Normal reflex, +++: Brisk reflex, ++++: Exaggerated reflex

Reflexes	Right	Left
Biceps reflex	++	++
Triceps reflex	++	++
Supinator reflex	Absent	Not assessable
Knee reflex	++	Absent
Achilles reflex	Not assessable	Not assessable
Plantar reflex	Extensor response	Flexor response

Assessment

Tone assessment in the upper and lower limbs indicated hypotonia, according to the tone grading scale as mentioned in Table 2. 

**Table 2:** Tone assessment in bilateral upper and lower limbs according to the tone grading scale 1+: Decreased response (hypotonia)

Side	Upper limb	Lower limb
Right	1+	1+
Left	1+	1+

Investigations

**Figure 2:**
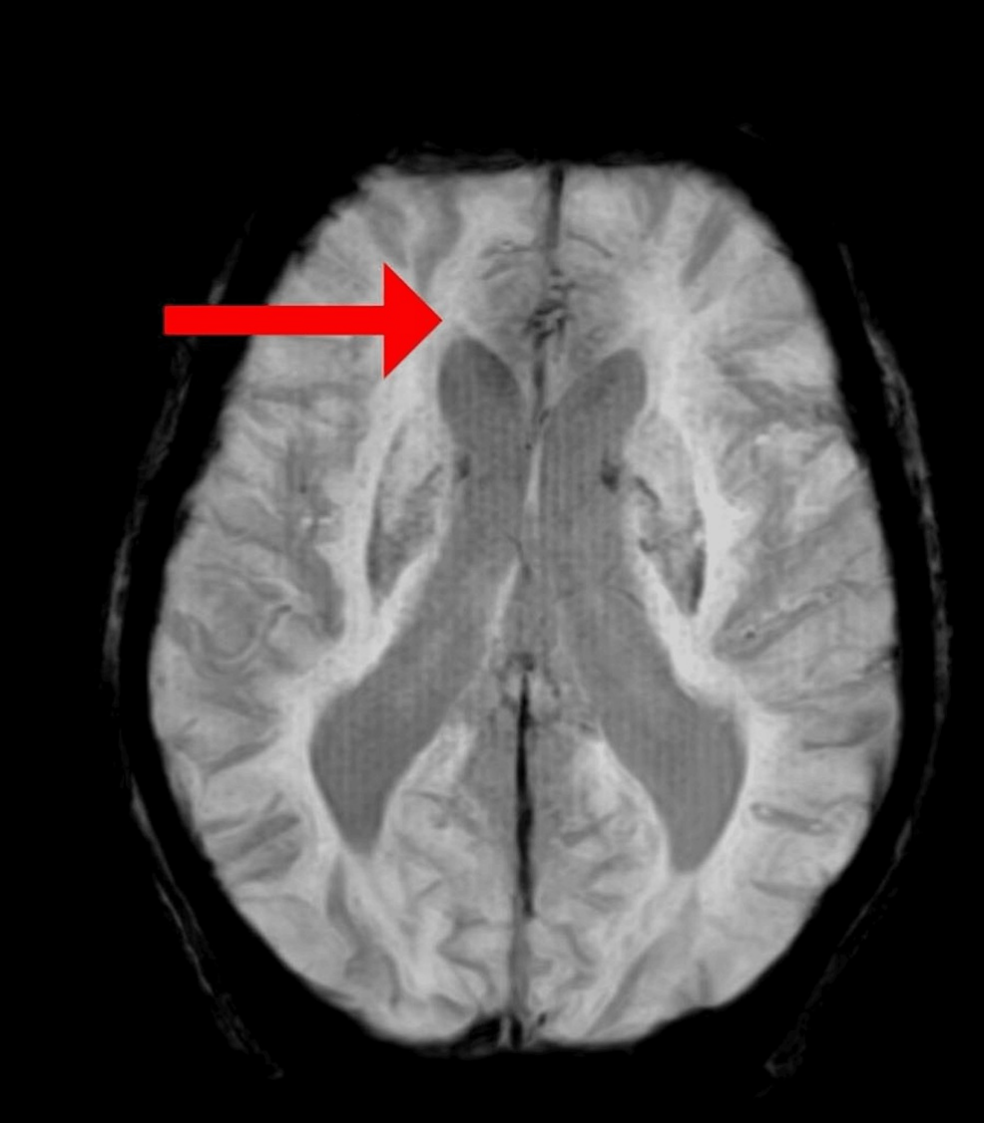
MRI showing bilateral periventricular deep white matter showing small vessel ischemic changes (red arrow)

MRI of the brain was done, which revealed bilateral periventricular deep white matter showing small vessel ischemic changes, as shown in Figure 2.

Physiotherapy management

A detailed physiotherapy protocol was planned that targeted the patient's symptoms. This protocol was planned for six weeks, six days per week. Depending on the severity of the symptoms, the treatment protocol was planned for better recovery. The patient's recovery was monitored, and accordingly, the progression of exercises was done, as mentioned in Table 3.

**Table 3:** Physiotherapy rehabilitation protocol SLT: Speech-language therapy; MIT: Melodic intonation therapy; PNF: Proprioceptive Neuromuscular Facilitation; D1: Diagonal pattern; ACBT: Active cyclic breathing technique; TRT: Task-Related Training; MRP: Motor Relearning Programme; CIAT: Constraint-Induced Aphasia Therapy; PACE: Promoting Aphasics Communication Effectiveness [11,13-17]

No.	Problem list	Goals	Interventions
1	Generalized weakness	To reduce symptoms of generalized weakness	Enhance the strength of each muscle group (strengthening exercises). Initially start with PNF D1 flexion and extension pattern for the upper limb and lower limb with hold relax technique. The ability to maintain one's weight when sitting and standing can be improved by MRP, task-specific training, TRT, and focus on progression to resisted exercises for weak muscles
2	Reduced range of motion and flexibility	To improve range of motion and flexibility	Weeks one and three mainly focus on active assisted and then, in weeks four and six, active ROM exercises and stretching
3	Reduced core strength	To improve core strength	Initially static abdominals, and then progress to abdominal curls, pelvic bridging
4	Impaired balance and coordination	To maintain good balance and coordination	Initially to improve balance: For static balance - weight shifts over both sides in sitting balance, for standing balance - tandem standing, Once static balance is achieved, the patient can progress to dynamic balance- multidirectional reach-outs for sitting balance, standing with shifting body weight side to side, tandem stance
5	Hemispatial visual neglect, other disorders such as depression and anxiety	To reduce the symptoms of hemispatial visual neglect and other conditions like depression and anxiety	Mirror treatment has been shown to improve motor skills along with impairment, daily living activities, pain, and visuospatial neglect in a variety of subjects. Action observation therapy - the Benson relaxation method (BRM) (10 minutes in the morning and evening to gain the health benefits associated with relaxation), touch-based skills reduce levels of anxiety and depression and can be initiated as early as possible to get better recovery
6	Excessive secretions in the chest	To clear the chest of excessive secretions	ACBT, postural drainage in lower zones, suctioning, and breathing exercises: diaphragmatic breathing, pursed lip breathing
7	Difficulty in articulating speech	To enhance articulation along with the tone and melody of speech	SLT (for roughly 30 to 60 minutes a day, two times a week, for a duration of six weeks), transcranial magnetic stimulation
8	Decreased verbal output in non-fluent aphasia	To improve verbal output in non-fluent aphasia	MIT for six weeks, with each session for about 30 to 40 minutes
9	Word retrieval difficulty	To improve word retrieval capacity	Word retrieval approach - in this, semantic and phonological hierarchical clues are used to stimulate the recovery of verbs and objects and then the intervention progresses to the formation of sentences
10	Paraphasias: difficulty in finding words, different comprehension affected at different levels, writing along with reading problems	To improve comprehension and alleviate writing and reading problems	The intense therapy model CIAT is predicated on making patients use verbal oral language as the only means of communication
11	Lack of good communication skills	To improve communication skills	PACE therapy

**Figure 3:**
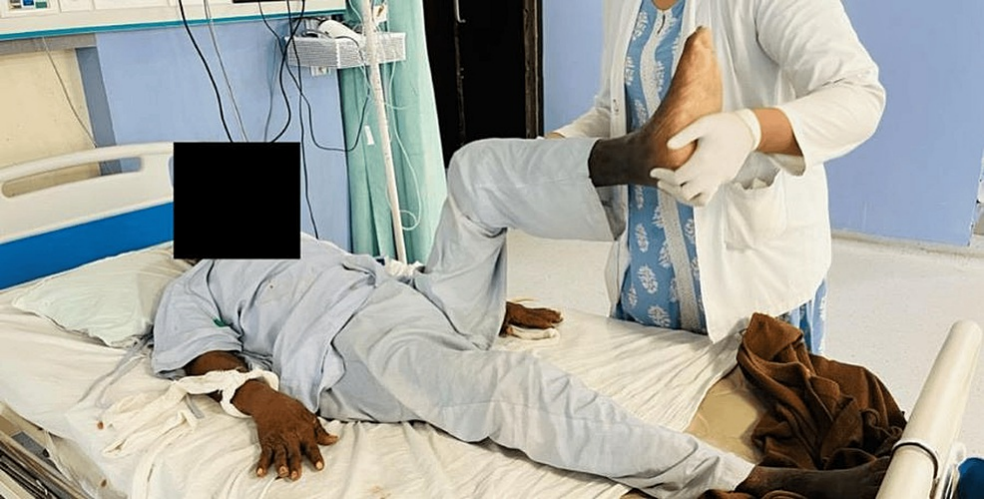
Patient performing active assisted lower limb exercises

In Figure 3, the patient is performing active assisted lower limb exercises.

**Figure 4:**
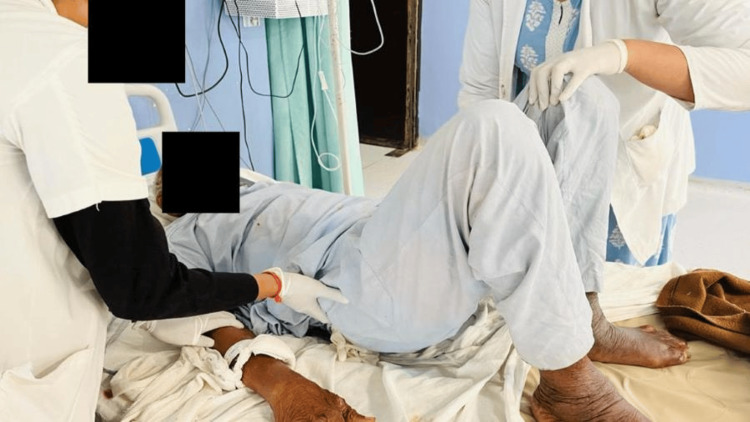
Patient performing active assisted pelvic bridging

In Figure 4, the patient is performing active assisted pelvic bridging.

**Figure 5:**
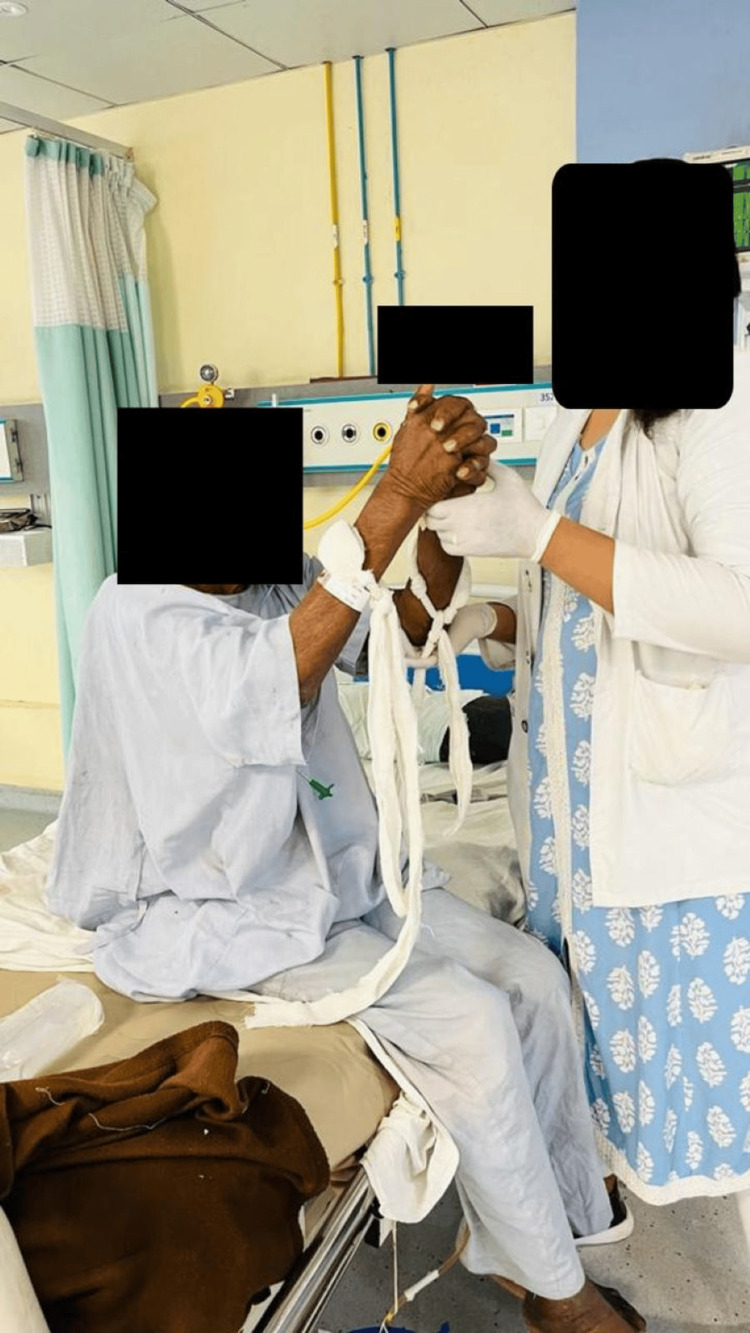
Therapist assisting in performing upper limb mobility exercises

Figure 5 depicts the therapist assisting in performing upper limb mobility exercises. 

**Figure 6:**
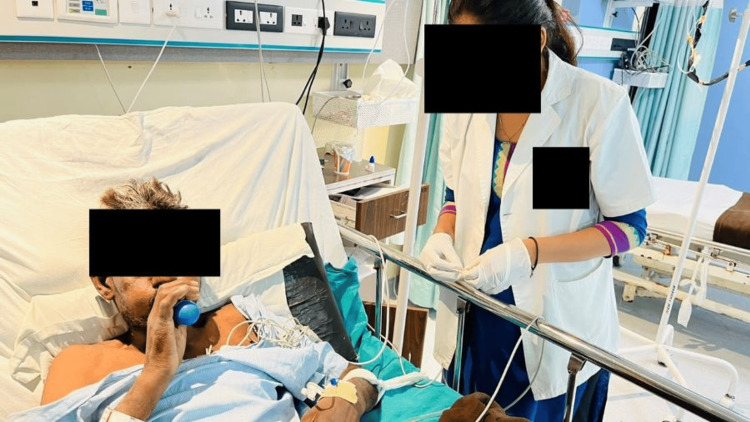
The patient was asked to perform balloon activities to improve the inspiratory capacity

The patient was asked to perform balloon activities to improve his inspiratory capacity (Figure 6). 

Outcome measures

Outcome measures like the ICU Mobility Scale, Functional Independence Measure, and Progressive Aphasia Severity Scale (PASS) were taken before, after treatment, and in a follow-up after three weeks of treatment to monitor the patient's recovery (Table 4). 

**Table 4:** Outcome measures

Outcome measure	Pre-intervention	Post-intervention	Follow-up (after three weeks of treatment)
ICU Mobility Scale	Score 0 - Nothing (lying in bed)	Score 3 - Sitting over the edge of the bed	Score 4 - The patient was able to stand with minimal assistance
Functional Independence Measure	Level 2 - Maximal assistance	Level 4- Minimal assistance	Level 5 - Supervision
Progressive Aphasia Severity Scale (PASS)	3 (severe impairment)	1 (mild impairment)	1 (mild impairment)

Manual muscle testing according to the Oxford scale is shown in Table 5.

**Table 5:** Manual muscle testing 2+: Full range of motion in a gravity-eliminated plane, breaks upon minimum resistance; 3: Full range of motion against gravity with no resistance

Joint	Pre-intervention	Post-intervention
Shoulder flexors	2+	3
Wrist flexors	2+	3
Hip flexors	2+	3
Hip extensors	2+	3
Knee flexors	2+	3
Knee extensors	2+	3
Ankle flexors	2+	3
Ankle extensors	2+	3

## Discussion

Broca’s aphasia occurs because of injury to the cortical language centre of the brain that is situated in the frontal lobe, on the left side, which is responsible for speech along with some motor movements [18]. Some drugs like catecholaminergic agents, acetylcholine esterase inhibitors, neurotrophic factors, transcranial magnetic stimulation, and transcranial direct stimulation can be used in managing patients with Broca's aphasia. Belin et al. have studied the recovery from non-fluent aphasia after melodic intonation therapy (MIT). It was found that subjects who had aphasia had good results with MIT [19]. Patients with tuberculosis may have lymphadenomegaly [20].

In the above case report, the patient complained of slurred speech, visual impairments, stiffness in the neck, seizures, and headache, for which reflex, tone, and some neurological signs of TBM (Brudzinski's sign) were positive. Outcome measures included the ICU Mobility Scale, Functional Independence Measure, and Manual Muscle Testing were used. In the investigation, an MRI of the brain was done. The case report details a comprehensive rehabilitation approach for non-fluent aphasia, encompassing physical therapy for muscle strength and stability, cognitive interventions like constraint-induced aphasia therapy (CIAT) for comprehension and communication, and speech-language therapy (SLT) for speech articulation. Balance exercises and mirror therapy were employed to address spatial awareness issues, while anxiety symptoms were managed with relaxation techniques. The integrated program aims to alleviate symptoms and enhance verbal output and language comprehension. All these above interventions used in this study are found to be more effective in improving the patient's QoL and overall well-being.

## Conclusions

The above case highlights the intricate relationship between TBM and Broca's aphasia, resulting in a myriad of neurological symptoms and some other complaints of headache, fever, and blurred vision leading to neurological deficits, seizures, and language impairment. An integrated physiotherapy approach encompassing various interventions including MIT, CIAT, Promoting Aphasics' Communicative Efficiency (PACE) therapy, SLT, and targeted exercises focusing on the patient's symptoms like weakness, articulation, balance, and coordination, had shown significant improvements in the patient's functional abilities and communication skills. Addressing not only the linguistic deficits but also associated physical weaknesses and functional limitations, this holistic rehabilitation approach led to improved mobility, speech articulation, comprehension, and overall communication effectiveness. In conclusion, the integrated physiotherapy rehabilitation program outlined in this case report exemplifies its efficacy in ameliorating the multifaceted impact of Broca's aphasia induced by TBM, thereby enhancing the patient's overall well-being and functional independence.
